# Strigolactones shape the assembly of root-associated microbiota in response to phosphorus availability

**DOI:** 10.1128/msystems.01124-23

**Published:** 2024-05-23

**Authors:** Pubo Chen, Pingliang Huang, Haiyang Yu, Huang Yu, Weicheng Xie, Yuehua Wang, Yu Zhou, Li Chen, Meng Zhang, Ruifeng Yao

**Affiliations:** 1 State Key Laboratory of Chemo/Biosensing and Chemometrics, Hunan University, Changsha, China; 2 Hunan Provincial Key Laboratory of Plant Functional Genomics and Developmental Regulation, Hunan University, Changsha, China; 3 College of Biology, Hunan University, Changsha, China; 4 Yuelushan Lab, Changsha, China; 5 School of Resource and Environment and Safety Engineering, University of South China, Hengyang, China; 6 Hunan Institute of Microbiology, Changsha, China; 7 Greater Bay Area Institute for Innovation, Hunan University, Guangzhou, China; University of Pretoria, Hatfield, South Africa

**Keywords:** strigolactones, phosphorus, *Arabidopsis thaliana*, microbial community, keystone taxa

## Abstract

Plants rely on strigolactones (SLs) to regulate their development and form symbiotic relationships with microbes as part of the adaptive phosphorus (P) efficiency strategies. However, the impact of SLs on root-associated microbial communities in response to P availability remains unknown. Here, root microbiota of SL biosynthesis (*max3-11*) and perception (*d14-1*) were compared to wild-type Col-0 plants under different P concentrations. Using high-throughput sequencing, the relationship between SLs, P concentrations, and the root-associated microbiota was investigated to reveal the variation in microbial diversity, composition, and interaction. Plant genotypes and P availability played important but different roles in shaping the root-associated microbial community. Importantly, SLs were found to attract *Acinetobacter* in low P conditions, which included an isolated CP-2 (*Acinetobacter soli*) that could promote plant growth in cocultivation experiments. Moreover, SLs could change the topologic structure within co-occurrence networks and increase the number of keystone taxa (e.g., Rhizobiaceae and Acidobacteriaceae) to enhance microbial community stability. This study reveals the key role of SLs in mediating root-associated microbiota interactions.

**IMPORTANCE:**

Strigolactones (SLs) play a crucial role in plant development and their symbiotic relationships with microbes, particularly in adapting to phosphorus levels. Using high-throughput sequencing, we compared the root microbiota of plants with SL biosynthesis and perception mutants to wild-type plants under different phosphorus concentrations. These results found that SLs can attract beneficial microbes in low phosphorus conditions to enhance plant growth. Additionally, SLs affect microbial network structures, increasing the stability of microbial communities. This study highlights the key role of SLs in shaping root-associated microbial interactions, especially in response to phosphorus availability.

## INTRODUCTION

The critical role of soil root microbiota in guaranteeing plant health and environmental sustainability has recently been highlighted by several studies (1–3). It is believed that co-evolutionary associations between rhizosphere microbial communities and plants have persisted for over 450 million years (4–6). Root exudates can bridge the communication gap between plant and soil microbes to enhance plant performance under abiotic stress (7). Plant root exudates such as strigolactones (SLs) can attract arbuscular mycorrhizal fungi (AMF) and nitrogen-fixing rhizobia, forming symbiotic relationships that enhance host nutrient acquisition (8, 9). Despite these findings, the precise impact of SLs on the root microbiome of non-mycotrophic plants such as *Arabidopsis thaliana* remains elusive.

SLs, a class of terpenoid secondary metabolites derived from carotenoids, are known to function as endogenous and rhizosphere signaling molecules (10, 11). The biosynthesis and perception pathway of SLs involve multiple proteins. In *A. thaliana*, Dwarf27, MORE AXILLARY GROWTH 3 (MAX3) MAX4, and MAX1 are involved in the biosynthesis of SLs, while MAX2 and DWARF14 (D14) are responsible for SL signaling (12, 13). SLs play a prominent role in regulating plant–microbe interactions by acting as rhizosphere signaling molecules, facilitating the recruitment of symbiotic microbes to alleviate abiotic stress in plants (14–16). Intriguingly, recent studies revealed that plants can release SLs into the rhizosphere soil, where they alter soil microbial composition and diversity (17). Notably, the biosynthesis and perception of SLs in rice have been observed to have a significant impact on the composition of both bacterial and fungal communities in the roots (18). These findings highlighted the multifaceted and complex roles of SLs in plant–microbe interactions and the need for further investigation into their mechanisms.

Phosphorus (P), an indispensable macronutrient for plant growth and development, is involved in crucial biochemical processes, including nucleic acid synthesis, enzyme regulation, and energy metabolism (19, 20). Although present in ample quantities in soils, its bioavailability is often restricted due to its complex inorganic and organic forms (21). Microbes play a crucial role in regulating plant productivity and are major contributors to the improvement of plant P uptake in ecological environments (22–24). Under P limitation conditions, plants respond to stress by increasing their secretion of SL, which is an adaptive strategy to acquire P by actively recruiting AMF (25). Additionally, the negative regulation exerted by PHR1 on certain components of the plant immune system would increase vulnerability to pathogenic invasion (26–28), as well as modifications to the plant microbiome under phosphate deprivation (29, 30). Besides, previous studies have revealed that the activating PHR-associated genes can trigger the expression of SL biosynthesis genes to influence the root microbiome (31, 32). Consequently, these findings provide new perspectives on how plants establish a mutually beneficial relationship with root-associated microbes in response to P-deficient environment.

This study aimed to clarify how the biosynthesis and perception of SL affect the assembly of root microbes in response to different P soil availability. We hypothesize that *A. thaliana* plants can selectively recruit beneficial microbes in the rhizosphere soil to adapt to P-deficient conditions by SL-mediated signaling. To test this hypothesis, we used SL biosynthetic (*max3-11*), perception (*d14-1*), and Col-0 plants to evaluate their effect on root microbial diversity, composition, and interaction under different levels of P. Sequencing was employed to analyze the alterations of root-associated microbiome. Our findings can provide insights into the underlying mechanisms between SLs and soil microbes to influence plant growth and development under different P conditions.

## MATERIALS AND METHODS

### Plant materials, growth conditions, and soil chemical analysis

**Fig 1:**
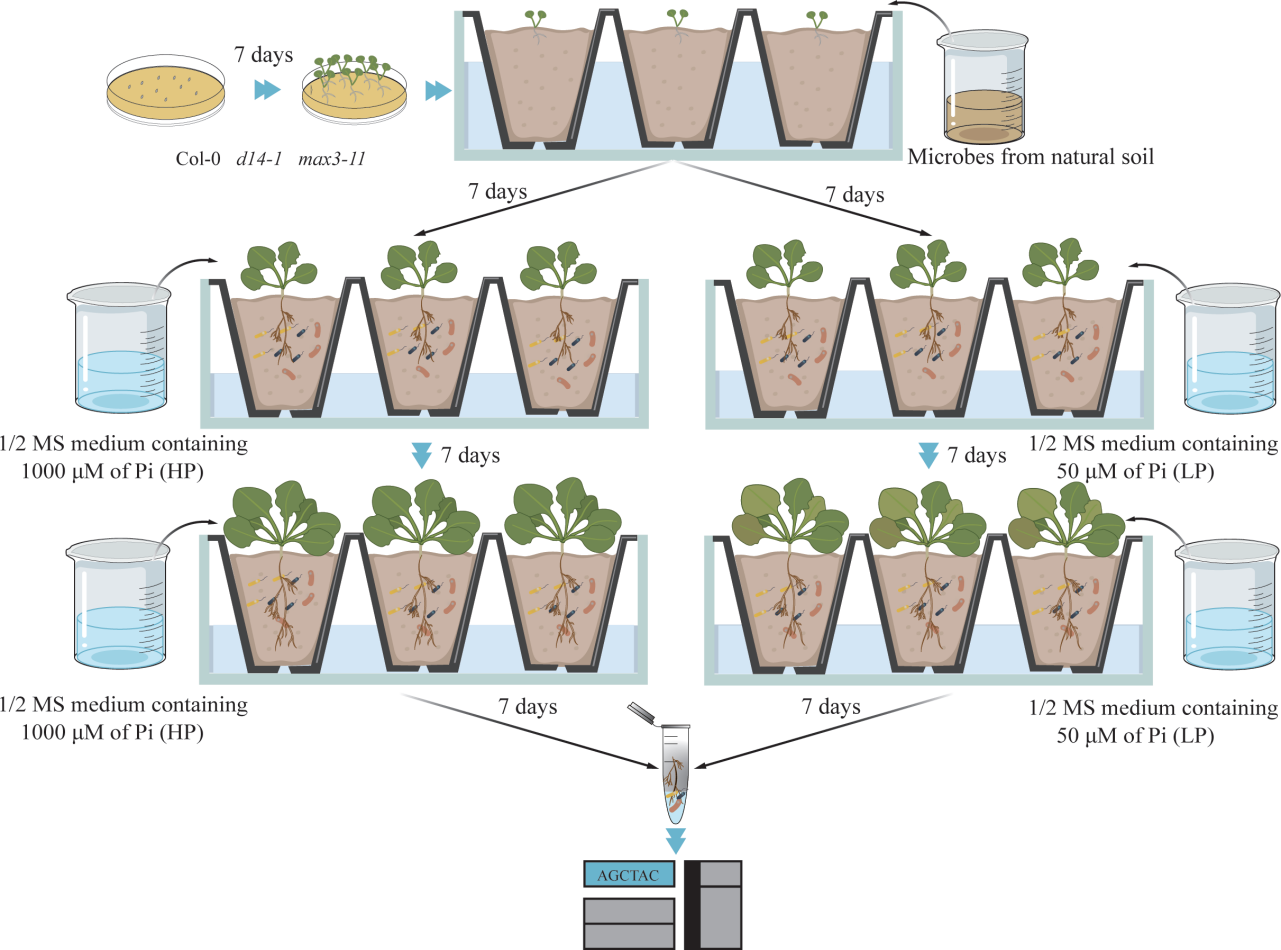
Experimental design for investigating the effect of SLs on root-associated microbial community in response to P availability. *A. thaliana* ecotype Col-0, *max3-11*, and *d14-1* mutant were grown in a mixture of vermiculite and sterilized commercial soil with natural microbial community. Arabidopsis plants were grown in 1/2 MS medium with 50 µM Pi and 1,000-µM Pi concentrations twice a week for total 21 days.

The *max3-11* mutant is deficient in SL biosynthesis due to a mutation in the MAX3 gene, which encodes an enzyme crucial for the production of SLs, while the *d14-1* mutant is defective in SL perception because of a mutation in the D14 gene, which encodes a receptor for SLs. Therefore, *A. thaliana* ecotype Col-0, SL biosynthesis (*max3-11*), and perception (*d14-1*) were used in this study. Seeds were surface sterilized with 20% bleach for 10 min and washed with sterile double-distilled water six times. Then seeds were planted on the half-strength Murashige and Skoog (1/2 MS) medium containing 0.7% agar and 1% sucrose. Sown seeds were chilled for 3 days at 4°C before being placed in a greenhouse under a photoperiod of 16 hours of light and 8 hours of dark for 21 days (light intensity of 120 µmol m^−2^ s^−1^, 22°C, and 60% relative humidity). To obtain a natural microbial community, we collected the top 10 cm of soil from a local forest farm in Changsha, Hunan Province, China (28°6.859′N, 113°3.583′E). Local soil pH was measured with pH electrode in a 1:2.5 soil:water suspension. Total carbon and total nitrogen in soil samples were determined on an element analyzer (Vario EL cube, Germany). Available P and available potassium were detected by molybdenum blue colorimetric method and flame photometry, respectively. Germinating seedlings were transplanted to a mixture of homogenized forest soil, vermiculite, and sterilized commercial soil (Klasmann-Deilmann, Germany) and (1:3:1) with four plants per pot. For each genotype, six pots were prepared, with each pot containing four plants, resulting in a total of 24 plants per genotype. This setup was replicated for both low-phosphate (LP) and high-phosphate (HP) conditions that included a total of 144 plants across all genotypes and conditions, amounting to 36 pots. The bulk soil samples were collected from the non-rhizosphere soil of plants grown in pots. Each of the four *Arabidopsis* genotypes was first grown in soil and watered with distilled water twice a week. Then plants in a pot were fertilized with 25-mL 1/2 MS medium at 50 µM Pi (LP) and 1,000 µM Pi (HP) concentrations twice a week and watered twice per week with distilled water (Fig. 1). After 21 days of growth, pots were photographed, and plant fresh weight was recorded immediately.

### Leaf anthocyanin measurement

Total leaf anthocyanin measurement was adapted from (33). Briefly, 50–100 mg of leaf tissue homogenized thoroughly with a pestle were cultured in 300 µL of extraction buffer (methanol solution containing 1% HCl) overnight at 4°C. Subsequently, the homogenized leaf tissue was extracted by adding 200 µL of distilled water followed by an equal volume of chloroform for phase separation. The content of anthocyanins was measured on a 96-well OptiPlate microplates (PerkinElmer, USA) of the aqueous phase (A530–0.25  ×  A657) and normalized to the fresh weight. Three independent biological samples were performed for each genotype.

### APase activity and reactive oxygen species measurements

To obtain more accurate information on the response of the three genotypes to high and low P conditions, acid phosphatase (APase) activity and hydrogen peroxide (H_2_O_2_) were assessed in whole plants. These plants were grown from seedlings that had been cultivated on agar plates for 12 days. To this end, a liquid nitrogen-based grinding approach was employed, followed by quantification of 50 mg of the resulting frozen powder. The p-nitrophenyl phosphate hydrolysis assay was utilized, and spectrophotometric measurements were obtained at 405 nm to determine the activity. The outcomes were expressed as mU/mg protein, ascertained using the Bradford protein assay to determine the total protein content.

The levels of H_2_O_2_ were determined through histochemical analyses using a staining method that involved 1-mg/mL 3,3′-diaminobenzidine, as per established protocols (34). The amount of H_2_O_2_ present was calculated based on the absorbance change of the 415-nm titanium peroxide complex. To quantify the absorbance values, a standard curve was generated from known concentrations of H_2_O_2_. Three independent biological samples (eight to ten plants per sample) were performed for each genotype.

### DNA extraction, PCR amplification, and sequencing analysis

At 21 days of plant growth, we collected samples from each genotype under both HP and LP conditions for subsequent 16S rRNA amplicon sequencing. This means that for each genotype, a total of 12 samples were collected (six LP treatment samples and six HP treatment samples). DNA extraction from rhizosphere soil was performed as previously reported (35). Specifically, the roots were gently removed from the pots, and the loosely attached soil particles were shaken off, after which the roots were placed into a 50-mL sterile tube containing 10 mL of phosphate-buffered saline. The roots were treated with a tube rotator for 20 min to strip soils attached to the root surface, and the rhizosphere soils were obtained by centrifuging at 4,000 g for 15 min. The tube was rapidly frozen in liquid N and stored at −80°C until processing. DNA extractions were performed from the bulk soil and rhizosphere soil samples with six replicates using FastDNA SPIN Kit for Soil (MP Biomedicals). The extracted DNA was quantified using a NanoDrop ND-2000 spectrophotometer and then diluted into 2 ng µL^−1^ for subsequent PCR amplification. The bacterial and fungal amplicons originated from the same sample. The 16S rRNA gene (V3 and V4 regions) and the fungal ITS1 region were then amplified by the primer set 338F (5′-ACTCCTACGGGAGGCAGCA-3′), 806R (5′-GGACTACHVGGGTWTCTAAT-3′), ITS1-F (5′-CTTGGTCATTTAGAGGAAGTAA-3′), and ITS2 (5′-GCTGCGTTCTTCATCGATGC-3′) (36). The libraries were sequenced on the HiSeq 2500 Platform (Illumina, CA, USA) using a 2 × 250-bp kit in Shanghai Majorbio Biopharm Technology Co., Ltd. DADA2 microbiome pipeline was used to denoise the raw sequencing reads, resulting in individual reads referred to as amplicon sequence variants (ASVs) (37). Specifically, bacterial 16S rRNA gene sequences were truncated at 240 bp, and fungal ITS sequences were truncated at 200 bp, based on the quality score threshold of Q20, ensuring that only high-quality, reliable sequences were retained for analysis. Taxonomic assignment of ASVs was performed using the naive Bayes classifier method based on the silva_138 database for bacterial and fungal ITS sequence data using the default parameters based on the UNITE database (v8.0 dynamic release) (38, 39).

### Network construction and analysis

To remove the lower detected ASVs, we kept the average relative abundance of ASVs over 0.01% across all samples for subsequent microbial network analysis (40). Microbial networks were constructed by using the FastSpar (v1.0) algorithm (40), which can implement interaction networks to compute correlation values from compositional data. Fastspar was run using 50 iterations of 1,000 bootstraps to calculate the correlations. Only statistically significant (*P* < 0.01) correlations were used to infer the bacterial and fungal network, which was subsequently visualized using Gephi 0.9.2.

We utilized the R package igraph (https://github.com/igraph) to calculate these critical topological features through fast greedy optimization. Network analysis was designed to show possible microbial interactions and responses to environmental cues and identify its keystone species. The topological roles of nodes in the root microbiome were evaluated based on their within-module connectivity (Zi) and among-module connectivity (Pi) values: peripherals, connectors, module hubs, and network hubs. Nodes were categorized as module hubs (Zi > 2.5, Pi ≤ 0.62), connectors (Zi ≤ 2.5, Pi > 0.62), network hubs (Zi > 2.5, Pi > 0.62), and peripherals (Zi < 2.5, Pi ≤ 0.62), except for peripherals, and all other categories were considered potential keystone taxa due to their significant roles in network topology.

### Microbial community stability analyses

To elucidate the effects of SLs on the stability of microbial networks, we employed multiple metrics to assess the ecological network’s robustness, vulnerability, and cohesion using the R package “vegan.” Specifically, we computed the abundance-weighted mean interaction strength of each node to investigate the impact of species removal on the remaining species following Deng et al. (41). To assess network robustness, we subjected the network to simulations in which 50% of the nodes were randomly removed, repeating this process 100 times as described in Yuan et al. (42) (42). Additionally, we evaluated the speed of disturbance propagation within the network by computing the global efficiency, defined as the average efficiency of all possible pairs of nodes. Finally, we quantified the vulnerability of each node by determining the maximal vulnerability within the network, which reflects the node’s relative contribution to global efficiency (41).

We also calculated cohesion using both positive and negative values to quantify the connectivity of microbial communities and reveal the associations among taxa attributed to species interactions, as well as similarities and differences in their niches. We first computed an abundance-weighted matrix based on pairwise correlations across taxa. Next, we performed a “taxa shuffle” null module-correcting step and computed a connectedness matrix with average positive and negative correlations using a Pearson correlation test and 200 randomizations. Finally, we calculated positive and negative cohesions for each sample. For specific details about the computational procedure, please refer to Herren et al. (43).

### Isolation and identification of bacteria and growth-promoting experiments on Arabidopsis seedlings

Rhizosphere soil samples from Col-0 plants were serially diluted and spread on Luria-Bertani (LB) agar medium and nutrient agar media. After incubation at 28°C for 48 h, single colonies were picked and streaked for purification. Bacterial isolates were obtained from both HP and LP conditions. Then, the isolated bacterial strains were subjected to phosphate-solubilizing ability assays to confirm their potential as phosphate-solubilizing bacteria. The bacterial strains were inoculated on Pikovskayas agar plates containing insoluble tricalcium phosphate as a sole P source, and the formation of clear halo zones around the bacterial colonies was considered phosphate-solubilizing isolates. Finally, the genomic DNA of the purified phosphate-solubilizing bacteria was used to amplify 16S rRNA by using the universal primers 27F (5′-AGAGTTTGATCMTGGCTCAG-3′) and 1492R (5′-GGTTACCTTGTTACGACTT-3′). The PCR products were purified and sequenced, and the obtained sequences were compared with the National Center for Biotechnology Information (NCBI) database using the BLAST algorithm. The bacterial species were identified based on their highest similarity to the reference strains. Phylogenetic trees were constructed using the neighbor-joining method in the MEGA 11 software.

Seven-day-old *Arabidopsis* seedlings (*Col-0*, *d14-1*, and *max3-11*) were obtained following the abovementioned method. Subsequently, 12 plants were transferred to 1/2 MS agar plates supplemented with two different concentrations of Pi (50 µM and 1,000 µM). The phosphate-solubilizing strain was activated by shaking it in LB liquid mediums, and the cultures were then washed twice with sterile 10 mM MgCl_2_. After adjusting the OD_600_ to 0.1, the strain was spread on the agar plates prior to the transfer of the plants. Fresh weight and root length were measured after 12 days of *in vitro* culture conditions. We repeated this experiment three times for accuracy.

### Statistical analysis

To assess the diversity and composition of the root microbial communities, we employed Mothur v1.30.1 to calculate the Shannon index based on ASV information (https://github.com/mothur/). To visualize the overall patterns of microbial communities, we conducted principal coordinate analysis (PCoA) based on Bray–Curtis dissimilarity using the Vegan v2.5.3 package (44). To test for differences in community dissimilarities, we utilized the analysis of multiple-response permutation procedure (MRPP), analysis of similarity (ANOSIM), and permutational multivariate analysis of variance (PERMANOVA) with the VEGAN package in R (43). In order to identify potential microbial bioindicators in response to SL effects, we performed linear discriminant analysis (LDA) and linear discriminant analysis effect size (LEfSe) analyses (45). To test for significance among comparisons, we used Tukey’s tests with GraphPad Prism 8 (GraphPad Software Inc., San Diego, CA, USA) through one-way ANOVA.

## RESULTS

### Soil physicochemical properties and the effect of low P stress on plant growth

The soil physicochemical analysis in Table S1 provides the general information on local forest soil properties in this study. To better understand plant growth in both LP and HP conditions, we compared shoot fresh weight and anthocyanin accumulation to characterize the P stress response of plant (Fig. S1a). After 21 days of growth, fresh weight of all plant genotypes was significantly lower in plants grown under low P conditions than P-sufficient conditions (Fig. S1b). Similarly, Arabidopsis plants showed significant increased accumulation of anthocyanin under low P conditions (Fig. S1c).

### APase activity and reactive oxygen species content response to different concentrations of P

The results of APase activity and H_2_O_2_ content analysis in whole plants under HP and LP conditions are shown in Fig. S2. Col-0 plants showed the significant higher (*P* < 0.05) APase activity and H_2_O_2_ content among the three genotypes under HP conditions, whereas the APase activity of *max3-11* plants showed the lowest (*P* < 0.05) compared to others under LP conditions. Overall, our results suggested that Col-0 wild-type plants are more tolerant to low P compared to the other two genotypes.

### Effect of SLs on diversity and structure of microbial communities

**Fig 2:**
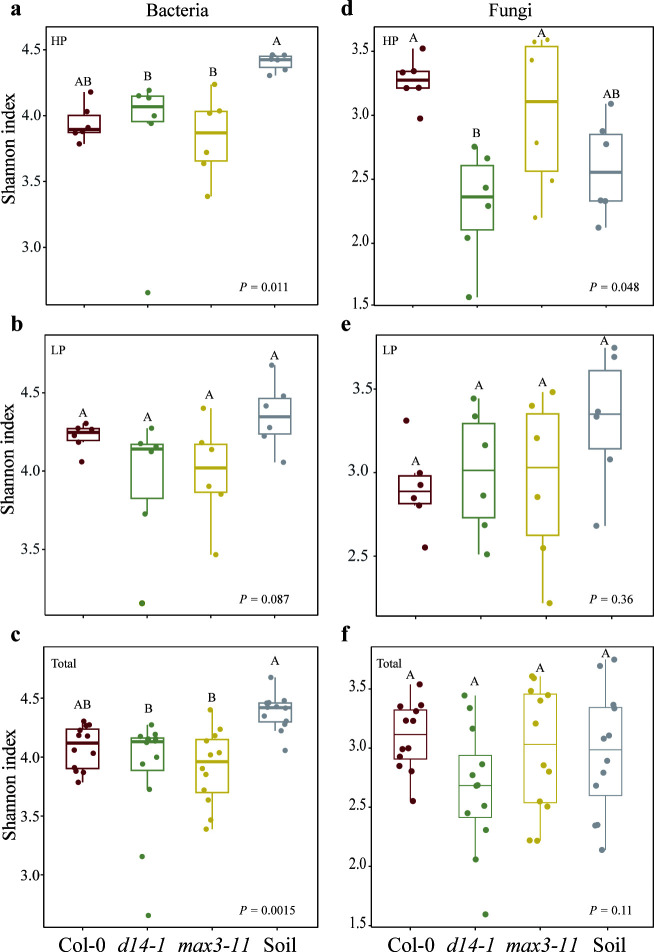
Variation in the alpha diversity (Shannon index) of bacteria (**a, b, c**) and fungi (**d, e, f**) communities from rhizosphere and bulk soil under LP and HP conditions. Data are the mean ± standard error (*n* = 6). Bacterial and fungal samples were both derived from the same soil samples across different treatments. Different lowercase letters over columns indicate significant differences (ANOVA, Tukey’s HSD test; *P* < 0.05).

**Fig 3:**
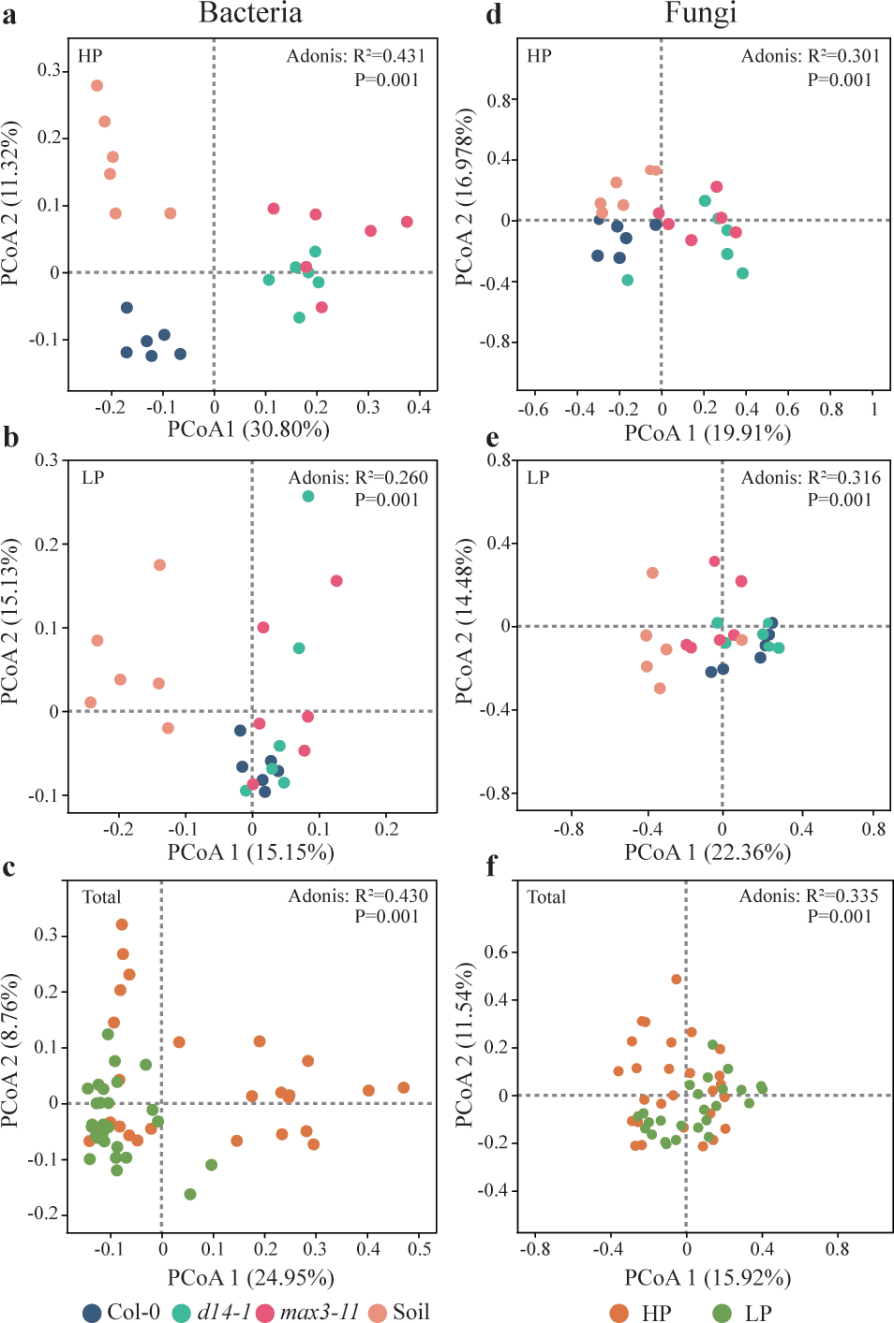
PCoA of bacteria (**a, b, c**) and fungi (**d, e, f**) based on the Bray–Curtis distance from rhizosphere and bulk soil under LP and HP conditions. Bacterial and fungal samples were both derived from the same soil samples across different treatments.

We analyzed the rhizosphere soil bacterial and fungal communities of three Arabidopsis genotypes related to SL signaling by sequencing 16S rRNA and ITS gene amplicons to determine any potential variations in microbial diversity. A significantly lower-bacteria Shannon diversity was observed in the mutants than in bulk soil. Additionally, we observed that the Shannon index of bacteria and fungi under low P conditions did not differ between Col-0 and mutants, while the d14-1 plants had a significantly lower Chao1 index than Col-0 plants in bacteria, suggesting a potential role of the D14 gene in shaping the bacterial alpha diversity under P-deficient conditions (Fig. 2; Table S2). The PCoA analysis showed that microbial communities from different plant genotypes were separated from bulk soil (*P* < 0.05, Adonis test), while the fungal communities associated with *max3-11* plants did not exhibit a clear separation from those of other genotypes (Fig. 3). Except for d14-1 plants of fungi communities in LP condition, three non-parametric tests (MRPP, PERMANOVA, and ANOSIM) showed consistently signiﬁcant (*P* < 0.05) difference in bacteria and fungi communities compared with Col-0 plants (Table 1). It suggested that SL mutant plants would affect the microbial community structure, while P-deficient conditions were also a key driver of microbial community structure.

**TABLE 1:** Dissimilarity tests of root-associated microbial communities based on three non-parametric tests

			MRPP		ANOSIM		PERMANOVA	
			Delta	*P*	*R*	*P*	*F*	*P*
Bacteria	HP	Col-0 vs *d14-1*	0.339	0.001	0.703	0.003	5.718	0.002
		Col-0 vs *max3-11* Col-0 vs soil	0.369 0.380	0.001 0.002	0.781 0.469	0.003 0.002	6.711 3.357	0.003 0.001
	LP	Col-0 vs *d14-1*	0.318	0.003	0.294	0.002	1.963	0.004
		Col-0 vs *max3-11* Col-0 vs soil	0.326 0.348	0.004 0.004	0.352 0.759	0.004 0.003	1.821 4.411	0.004 0.003
Fungi	HP	Col-0 vs *d14-1*	0.617	0.008	0.416	0.008	2.986	0.002
		Col-0 vs *max3-11* Col-0 vs soil	0.569 0.655	0.006 0.011	0.537 0.218	0.004 0.015	3.441 2.099	0.006 0.021
	LP	Col-0 vs *d14-1*	0.526	0.211	0.096	0.181	1.301	0.018
		Col-0 vs *max3-11* Col-0 vs soil	0.607 0.543	0.031 0.003	0.018 0.533	0.031 0.004	1.645 3.689	0.043 0.005

### Effect of SLs on the composition of microbial communities

**Fig 4:**
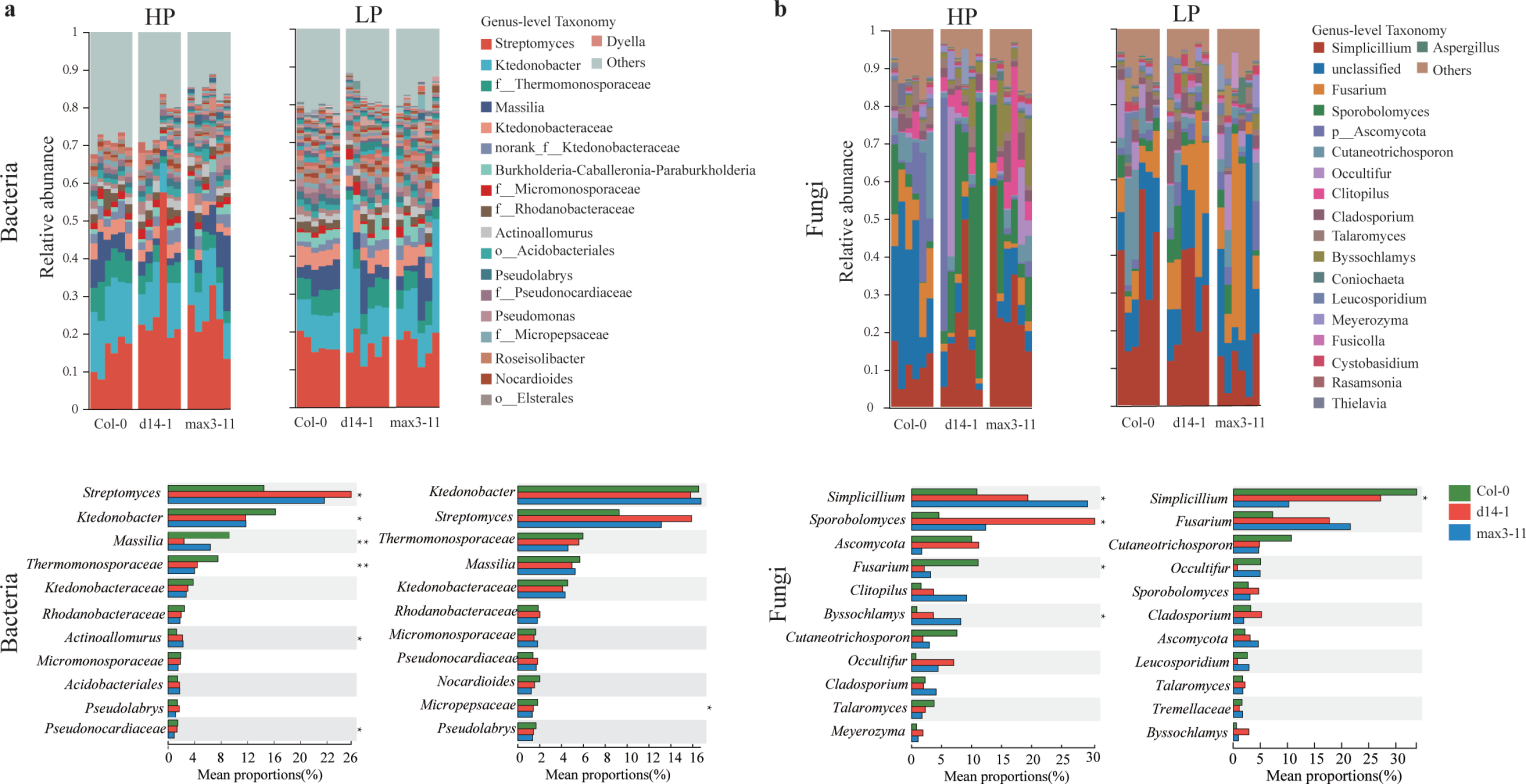
Top 20 relative abundances of the major genus of rhizosphere microbial communities under HP and LP conditions. (**a**) Rhizosphere bacteria, (**b**) rhizosphere fungi. The *x* axis represents the average relative abundance (*n* = 6), and the columns of different colors represent different genotypes (**c, d**). * Indicated significant difference among wild-type Col-0 and SL mutants *d14-1* and *max3-11* (ANOVA, Tukey’s HSD test; *P* < 0.05).

In this study, we investigated the changes in the composition of fungi and bacteria in the root of the three plant genotypes. Specifically, the bacterial communities consisted mainly of Actinobacteria (34.26%), Proteobacteria (27.25%), Chloroﬂexi (22.62%), Acidobacteria (4.44%), Bacteroidota (3.00%), and Gemmatimonadetes (1.57%). The fungal communities were predominantly composed of phyla Ascomycota (59.49%), Basidiomycota (25.19%), and Chytridiomycota (2.67%) (Fig. S3). Additionally, ternary plots showed that the composition of bacterial and fungal taxa of the *d14-1* and *max3-11* closely resembled that of Col-0, with only low numbers of ASVs being differentially abundant between these two mutants. Furthermore, we observed significant (Tukey’s honestly significant difference [HSD] test; *P* < 0.05) variation in both bacterial and fungal communities in the HP soil (Fig. 4). At the genus level, the abundant genera across all samples were *Streptomyces*, *Ktedonobacter*, and *Massilia*, while the most abundant fungal genera were *Penicillium* and *Sporobolomyces*. Among these, the relative abundance of *Streptomyces*, *Penicillium*, and *Sporobolomyces* in SL mutant plants was significantly higher than Col-0 plants in the HP condition, while the relative abundance of *Penicillium* was significantly enriched in the Col-0 plants of LP condition (Fig. 4). The taxonomic composition exhibited significant alterations between Col-0 and SL mutant plants in response to HP conditions. We also examined the SL-induced specific bacterial and fungal taxa by LEfSe analysis, designed to find any potential biomarkers. Specifically, we observed a significant increase in the abundance of *Bacillus* and *Simplicillium* under low P conditions (Fig. S4), which were identified as putative bioindicators for low P conditions. Overall, our study highlights the potential of taxonomic analysis for identifying beneficial microbial taxa in complex ecological systems.

### Microbial network stability and their topological properties

**Fig 5:**
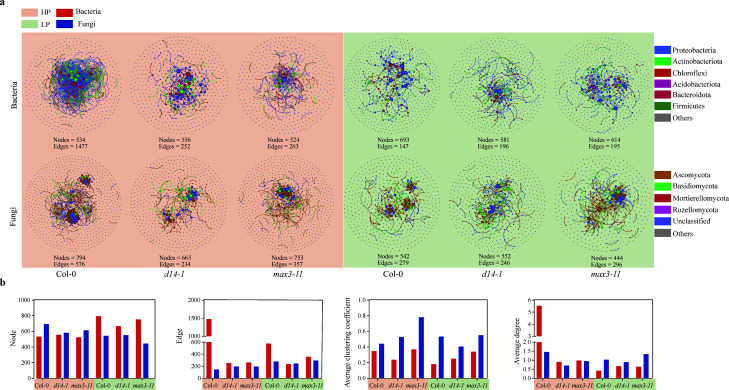
Visualization of constructed microbial networks in wild-type Col-0, SL mutant *d14-1*, and *max3-11* (**a**). Topological properties of the microbial co-occurrence networks in wild-type Col-0, SL mutant *d14-1*, and *max3-11* (**b**). With the node size proportional to node connectivity, the node color represents various phyla.

In order to evaluate the influence of SLs on the microbial interactions within the plant rhizosphere, we constructed correlation-based networks for the HP and LP conditions (Fig. 5). The topological and structural features of co-occurrence networks for microbial communities were found to be quite different between Col-0, *d14-1*, and *max3-11* plants. In particular, the number of total nodes ranged from 444 to 794, and the total edges ranged from 195 to 1477 (Table S3). Additionally, the clustering coefficients, average degree, and relative modularity of the Col-0 plants exhibited a trend toward higher than SL mutant plants. The above data indicated that microbial communities in Col-0 mutants exhibited a relatively complex network.

**Fig 6:**
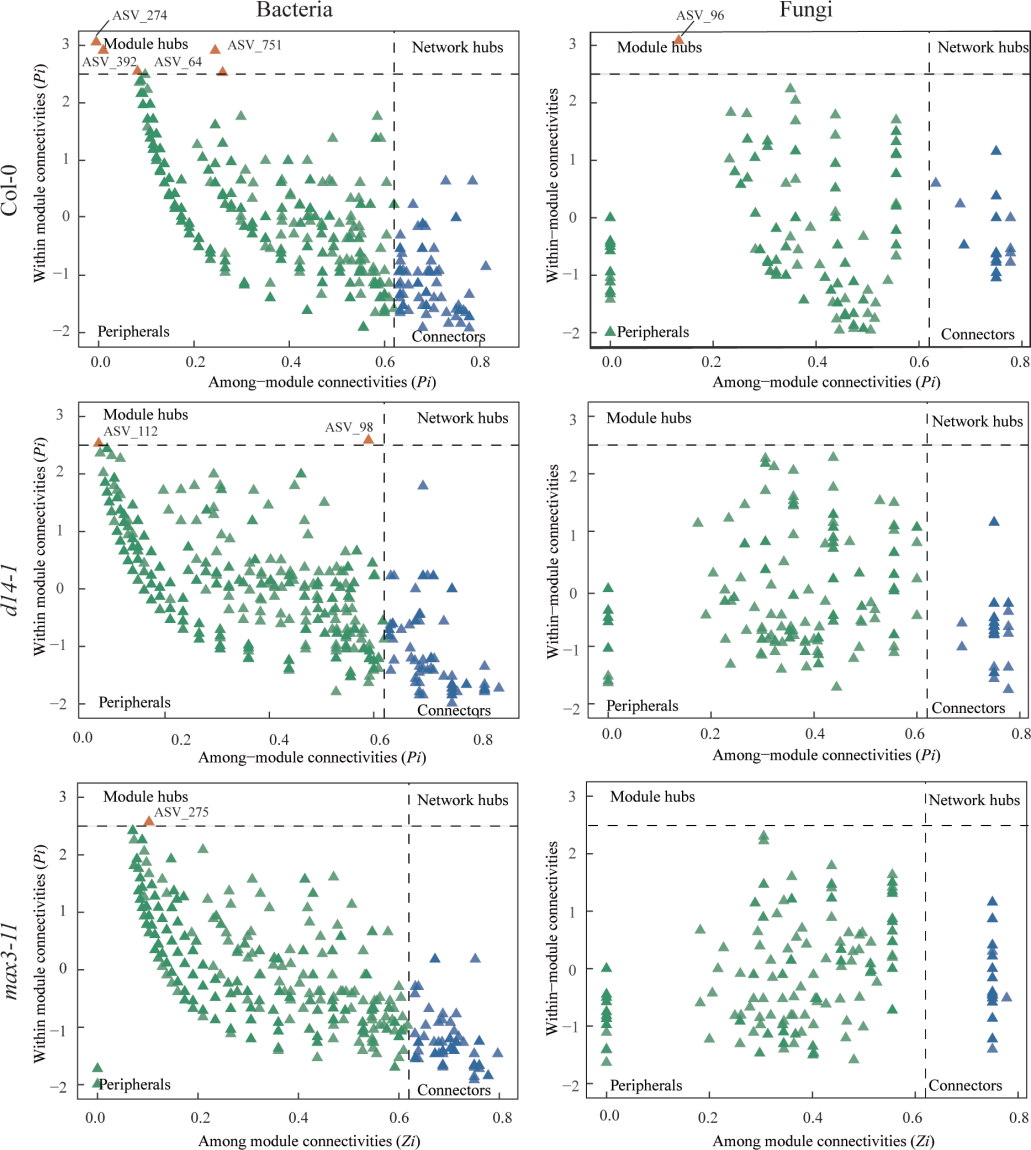
Identification of keystone taxa in wild-type and SL mutant *d14-1* and *max3-11* under LP conditions based on their topological roles in networks. Module hubs are identified as Zi ≥ 2.5, Pi < 0.62, connectors are identified as Zi < 2.5, Pi ≥ 0.62, and network hubs are identified as Zi ≥ 2.5, Pi ≥ 0.62.

Our results showed few differences in the proportion of connector nodes among Col-0 plants and SL mutant plants (Fig. 6; Fig. S5). In both HP and LP conditions, Col-0 plants exhibited a greater proportion of module hubs than SL mutant plants, indicating greater intramodular connectivity but reduced connectivity with nodes outside their respective modules. These bacteria keystone taxa in Col-0 plants at HP and LP conditions were ASV_186 (Acidobacteriaceae), ASV_1144 (Tepidisphaerales), ASV_64 (*Ktedonobacter*), ASV_274 (*Singulisphaera*), ASV_392 (Rhizobiaceae), and ASV_751 (*Terriglobus*), while the fungal keystone taxa were ASV_96 (unclassified). Our results indicated that SL-deficient plants affected the network structure and topological roles of individual ASVs and potential keystone taxa.

To assess how SL changes microbial network properties, we utilized robustness, vulnerability, and cohesion metrics to assess the stability of the microbial community across three plant genotypes. Our results indicate that under both HP and conditions, Col-0 plants exhibited significantly (*P* < 0.05) higher robustness than SL mutant plants (Fig. S6). Additionally, network vulnerability analysis revealed that Col-0 plants were more stable than SL mutant plants (Fig. S7). The negative:positive cohesion value was also higher in Col-0 plants, indicating a more stable microbial community (Fig. S8). Overall, these metrics provide strong evidence for the higher microbial community stability of Col-0 plants.

### Assessing the effects of functional bacteria on plant growth

**Fig 7:**
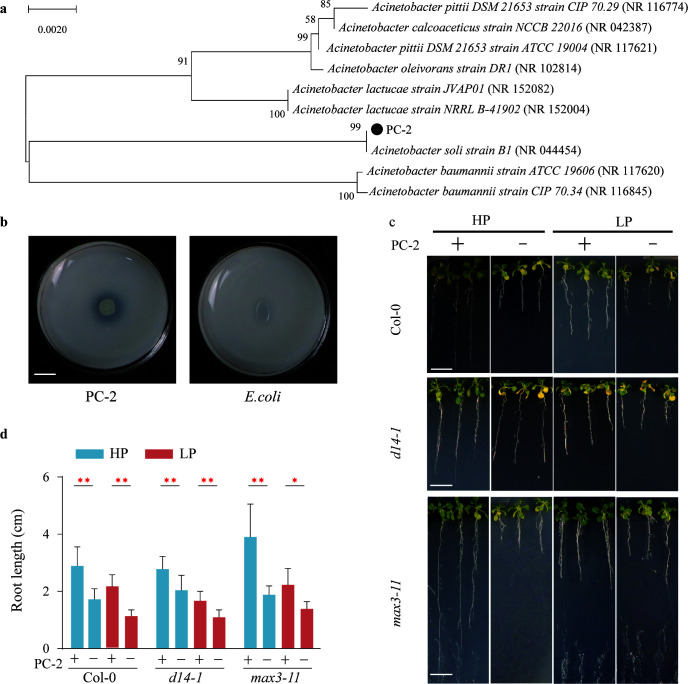
Effect of isolated bacteria on the growth of Arabidopsis seedlings under different P conditions. (**a**) 16S rRNA phylogenetic tree of bacterial strain CP-2. (**b**) Plant-beneficial trait was assayed in phosphate solubilization. (**c**) Growth of seedlings inoculated with the CP-2 under different P conditions. (**d**) Growth-promoting effect of CP-2 (*A. soli*) on the *Arabidopsis* seedlings. Data are the mean ± standard error (*n* = 30) (ANOVA and Tukey’s HSD test: *, *P* < 0.05; **, *P* < 0.01; ***, *P* < 0.001). The scale bar = 1 cm.

Although we have isolated 24 different morphologies of bacteria from the rhizosphere soil of Col-0 plants, less than 25% of the strains were capable of phosphate solubilization. Among them, CP-2 (*Acinetobacter soli*) showed a strong P-solubilizing ability. Thus, we selected this strain for subsequent co-cultural experiments (Fig. 7a through c). The high-throughput sequencing results indicated that the relative abundance of *A. soli* was more than 1% in the LP condition of Col-0 and significantly higher than other two plant genotypes (Fig. S9). The results showed that *A. soli* could significantly promote fresh weight and root lengths of Arabidopsis seedlings under LP and HP conditions (*P* < 0.05), while the change in HP condition of *max3-11* was *the most* apparent (Fig. 7d)*.* Collectively, our study has shed light on the potential implications of SLs in modulating the composition of the root-associated microbial community, with a particular focus on the recruitment of *A. soli* as a potential strategy for alleviating LP stress in plants. However, it is imperative to emphasize the necessity for further research to validate this conclusion.

## DISCUSSION

Unraveling the impacts of SLs on the root microbiota of *A. thaliana* is highly important to understand their ecological role in the plant–microbe interactions. We showed that plant genotypes and P availability play essential roles in shaping the root-associated microbial community. Remarkably, maintaining the microbial community stability may be primarily attributed to recruiting some beneficial microbes (e.g., Acidobacteriaceae, Rhizobiaceae, and *Acinetobacter*). These results supported our hypothesis that *A. thaliana* plants can selectively recruit beneficial microbes in the rhizosphere soil to adapt to P-deficient conditions via SL-mediated signaling.

Plant hormones play a crucial role in shaping the diversity and composition of the rhizosphere microbial community associated with plants (46–48). In this study, we did not observe differences in microbial alpha diversity among phenotypes under LP conditions, which is consistent with a previous study (49). It is well known that soil P content is a limiting factor that affects microbial diversity (50). The bacteria alpha diversity of SL mutant plants was significantly lower than bulk soil in the HP condition but not in the LP condition. One possible explanation is that the P deficiency environment had a greater influence on alpha diversity than the SL signaling pathway. Moreover, the PCoA analysis revealed distinct clustering patterns in plant microbial communities, with clear separation among bulk soil and SL mutant plants, indicating a significant effect of SL on the microbial community structure. Similar results were found in previous studies of rice root-associated microbiome (17), which demonstrated that both SL biosynthesis and perception were involved in regulating root microbial structure. It suggested that SL mutant plants would affect the microbial community structure, while P-deficient conditions were also a key driver of microbial community structure. In addition to the observed effects on microbial community composition and structure, it is noteworthy to highlight the high adaptability of Col-0 plants under low P conditions compared to the other two genotypes.

Although SLs were known to stimulate the germination of AMF spores and attract them to form mutualistic associations with the plant under P-deficient conditions (11, 51, 52), it is likely possible that SLs may attract other growth-promoting microorganisms to enhance nutrient acquisition for non-mycotrophic plants (16). Our key findings were that modifications to the SL biosynthesize and perception pathways affected the composition of rhizosphere bacterial and fungal communities. For example, we showed that SL mutant plants had significant differences in *Streptomyces*, *Ktedonobacter*, *Massilia*, *Penicillium*, and *Sporobolomyces* compared to wild-type Col-0. Moreover, our results showed that *d14-1* and *max3-11* plants increased the relative abundance of *Streptomyces* in both HP and LP conditions, which is consistent with the results of Arabidopsis mutants with jasmonic acid deficiency (53). Recently, it has been demonstrated that lipids and terpenoids with significant alterations exhibit increased accumulation in rice mutants d10 and d14. It might be partly due to the microbial composition variation in host selection pressure mediated by the host immune response and secondary metabolites among different plant genotypes (54–56). According to the LefSe analysis, the biomarker of *Massilia* and *Bacillus* was considered to be plant growth-promoting rhizobacteria, which can provide nutrients or signal substances for plants (57, 58). Collectively, our study has shed light on the potential implications of SLs in modulating the composition of the root-associated microbial community, with a particular focus on the recruitment of *A. soli* as a potential strategy for alleviating LP stress in plants. Several studies have shown that *Acinetobacter* spp. has the ability to solubilize P and improve plant growth under different environmental conditions (59, 60). The recruitment of plant growth-promoting rhizobacteria (PGPR) may be a critical mechanism for plants to cope with LP stress, and future investigations are required to determine if SLs can recruit specific microbial functions under such conditions directly. Overall, our study highlights the potential of taxonomic analysis for identifying beneficial microbial taxa in complex ecological systems.

However, microbial composition and diversity can only partially reveal the complexity of microbial interactions and address the effects of SLs on the stability of the root microbiota (61). Co-occurrence network analyses contribute greatly to investigating potential microbial community interactions (62, 63). Our findings suggested that *d14-1* and *max3-11* plants destabilized microbial community networks, resulting in less complexity and modularity. Also, a decrease in modularity within a microbial network may exacerbate network instability because of the higher prevalence of cross-module correlations among taxa (42, 64). For instance, plant–microbe mutualistic interactions, including plant growth-promoting rhizobacteria and phosphate-solubilizing microbes, may enhance each other by promoting the plant growth and development of their shared plant partners (65). Therefore, a low level of modularity in such interactions could weaken the potential for cooperation between bacteria and fungi. The ecological stability observed within the rhizosphere is attributed to the enhanced resource availability and niche habitability (66). These findings revealed that the microbial community is ecologically vital role in plant–microbe interactions.

Recent research suggested that highly connected taxa could potentially serve as potential keystone taxa, which play vital roles in the microbiome, and their removal can cause significant changes in the microbial community (67). Despite identifying keystone taxa in diverse environments, knowledge regarding keystone taxa in the *Arabidopsis* root microbiota still needs to be available. Our study found that keystone taxa in the Col-0 plants were increased under the LP conditions. This observation may be attributed to SL-mediated signaling recruitment of distinct keystone taxa to adapt to low P environments. For example, the keystone members within Acidobacteriaceae and Rhizobiaceae are important diazotrophs and PGPR (68). Additionally, due to their unique biotic interactions (as hubs or connectors), keystone taxa significantly contribute to microbial communities’ stability (69, 70). Our results demonstrated that the removal of keystone taxa from microbial networks significantly decreased network stability in Col-0 plants. Previous studies also reported that the removal of potential keystone taxa leads to a drastic shift in microbiome structure and functioning (70). Future synthetic community experiments and metagenomics analysis will aim at understanding the interaction of SL signaling and these keystone taxa in *Arabidopsis* plants.

Overall, this study clarified the effects of SLs on the rhizosphere microbial community of *Arabidopsis* plants. Root microbial communities were shaped by plant genotypes and P availability, which caused considerable microbial diversity and structure alterations in SL mutants. We found that SL could attract specific microbial species (CP-2) for plant adaptation to the P deficiency environment. Also, the microbial communities of SL mutant plants exhibited a reduced complexity and fewer keystone taxa, resulting in weaker interspecific interactions that could potentially compromise community stability. This study enhances the eco-evolutionary theory of non-mycotrophic plants influencing some specific microbiota through phytohormones SL.

## Data Availability

The 16S and ITS raw sequencing data can be found at the NCBI Sequence Read Archive (SRA) and the accession numbers PRJNA960720 and PRJNA966124. In this study, no new codes were developed, and the analysis tools employed were used with default settings, except where explicitly stated otherwise.

## SUPPLEMENTAL MATERIAL

Fig. S1 msystems.01124-23-s0001.tif Response of the *Arabidopsis* plants under Pi starvation.

Fig. S2 msystems.01124-23-s0002.tif Activity of total acid phosphatases and H_2_O_2_ concentrations.

Fig. S3 msystems.01124-23-s0003.tif Relative abundances of the dominant phyla of rhizosphere and bulk soil microbial communities.

Fig. S4 msystems.01124-23-s0004.tif LDA scores of the abundant taxa.

Fig. S5 msystems.01124-23-s0005.tif Identification of keystone taxa.

Fig. S6 msystems.01124-23-s0006.tif Robustness.

Fig. S7 msystems.01124-23-s0007.tif Vulnerability.

Fig. S8 msystems.01124-23-s0008.tif Negative:positive cohesion.

Fig. S9 msystems.01124-23-s0009.tif Relative abundance of *A. soli* in rhizosphere microbial communities of *A. thaliana*.

Supplemental material msystems.01124-23-s0010.docx Supplemental figure legends and tables.

ASM does not own the copyrights to Supplemental Material that may be linked to, or accessed through, an article. The authors have granted ASM a non-exclusive, world-wide license to publish the Supplemental Material files. Please contact the corresponding author directly for reuse.
