# Supplementary figures and images for: Strigolactones shape the assembly of root-associated microbiota in response to phosphorus availability
Source: mSystems. 2024 May 23;9(6):e01124-23. doi: 10.1128/msystems.01124-23 (PMC11237589; doi:10.1128/msystems.01124-23)

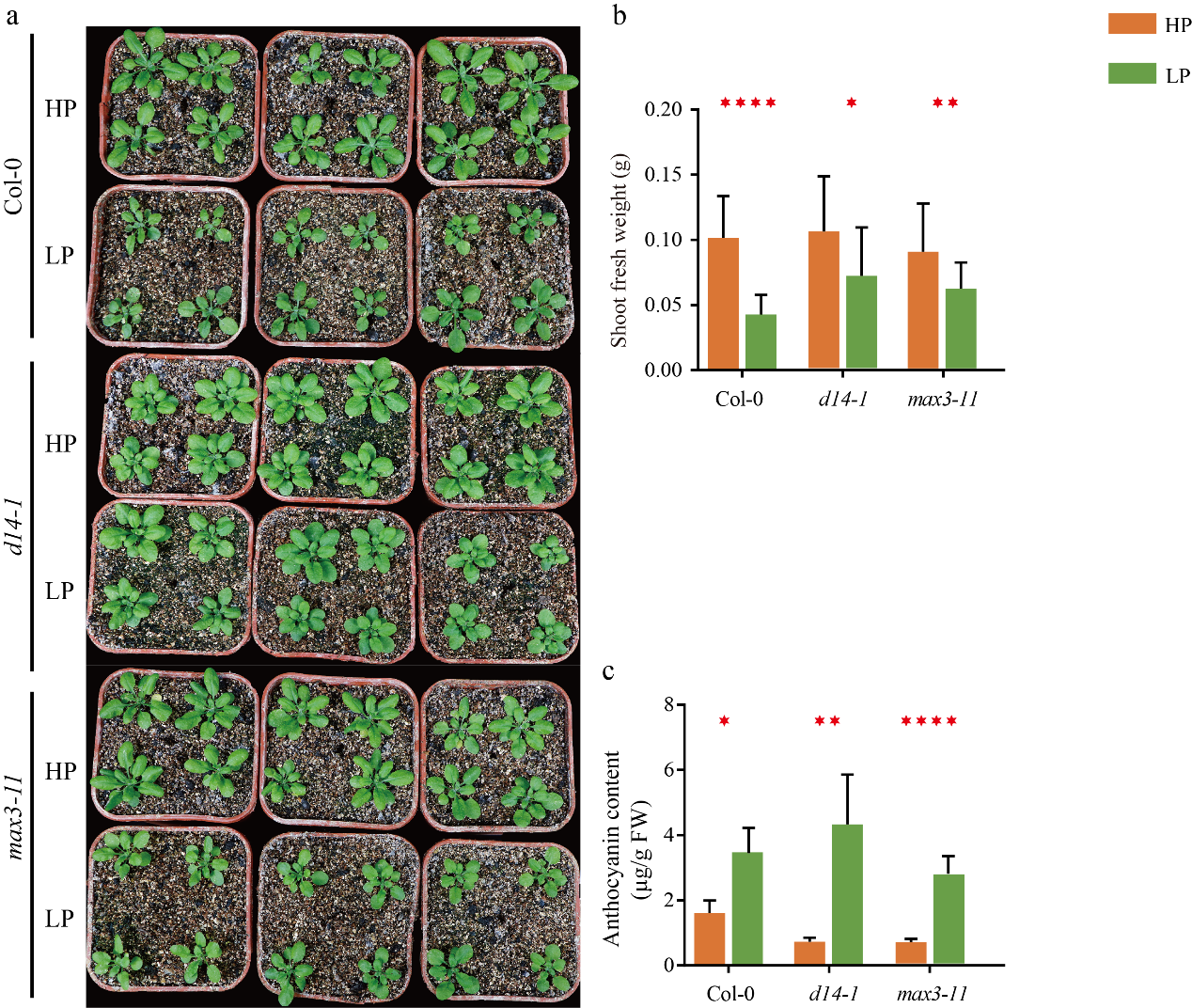

Supplement: Fig. S1 — Response of the Arabidopsis plants under Pi starvation. [file msystems.01124-23-s0001.tif]

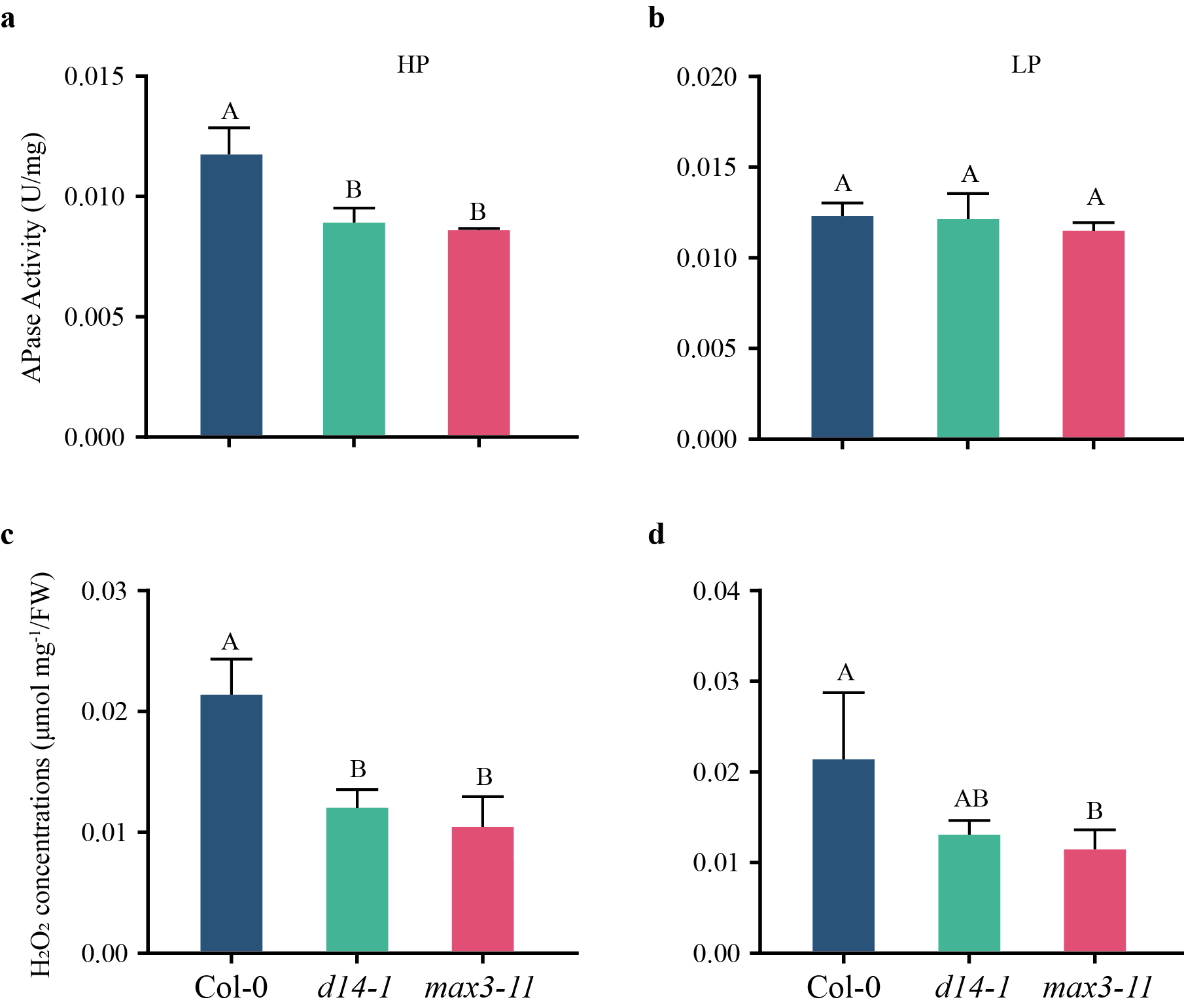

Supplement: Fig. S2 — Activity of total acid phosphatases and H2O2 concentrations. [file msystems.01124-23-s0002.tif]

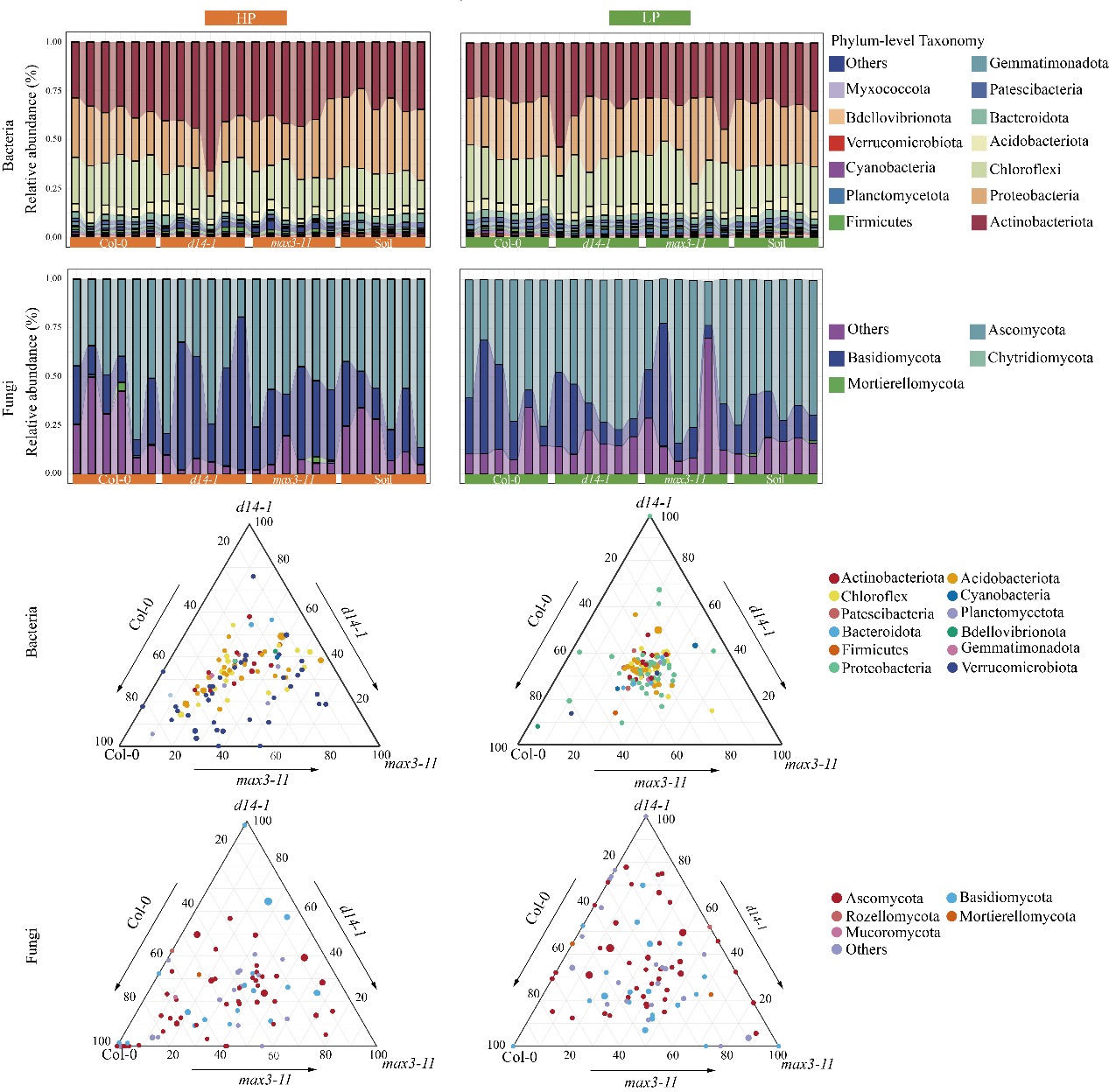

Supplement: Fig. S3 — Relative abundances of the dominant phyla of rhizosphere and bulk soil microbial communities. [file msystems.01124-23-s0003.tif]

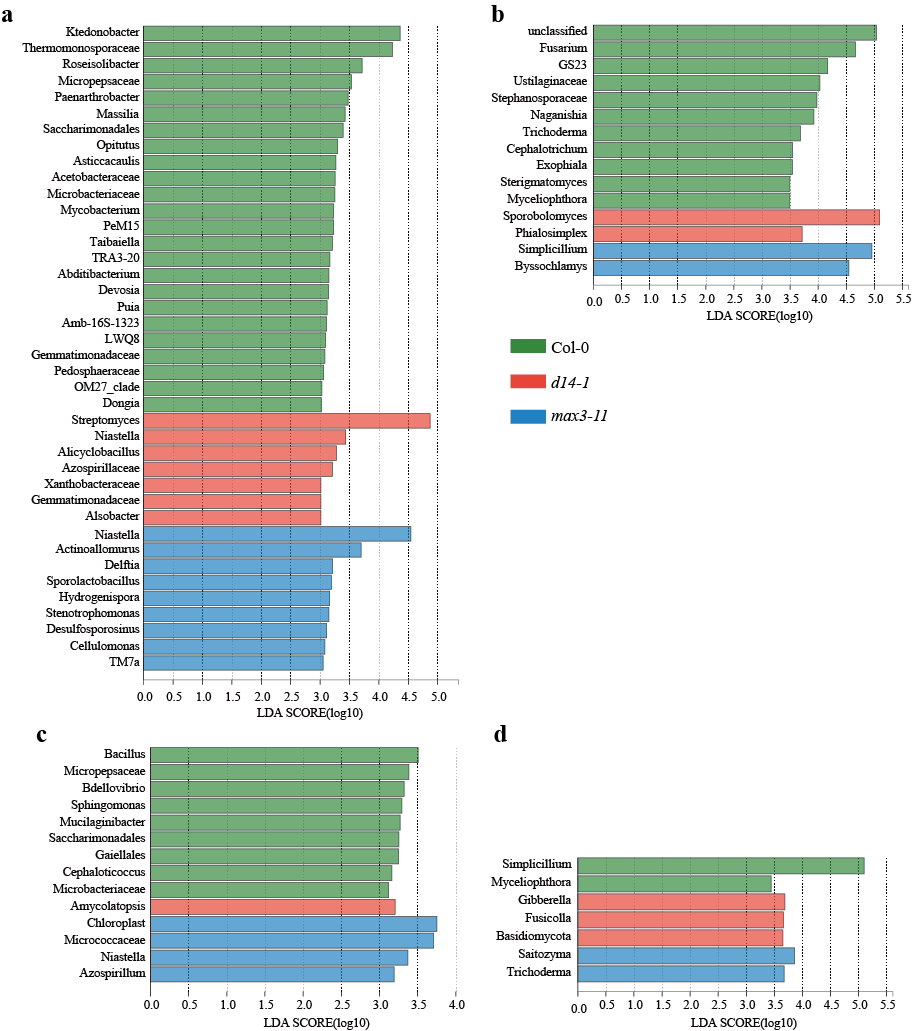

Supplement: Fig. S4 — LDA scores of the abundant taxa. [file msystems.01124-23-s0004.tif]

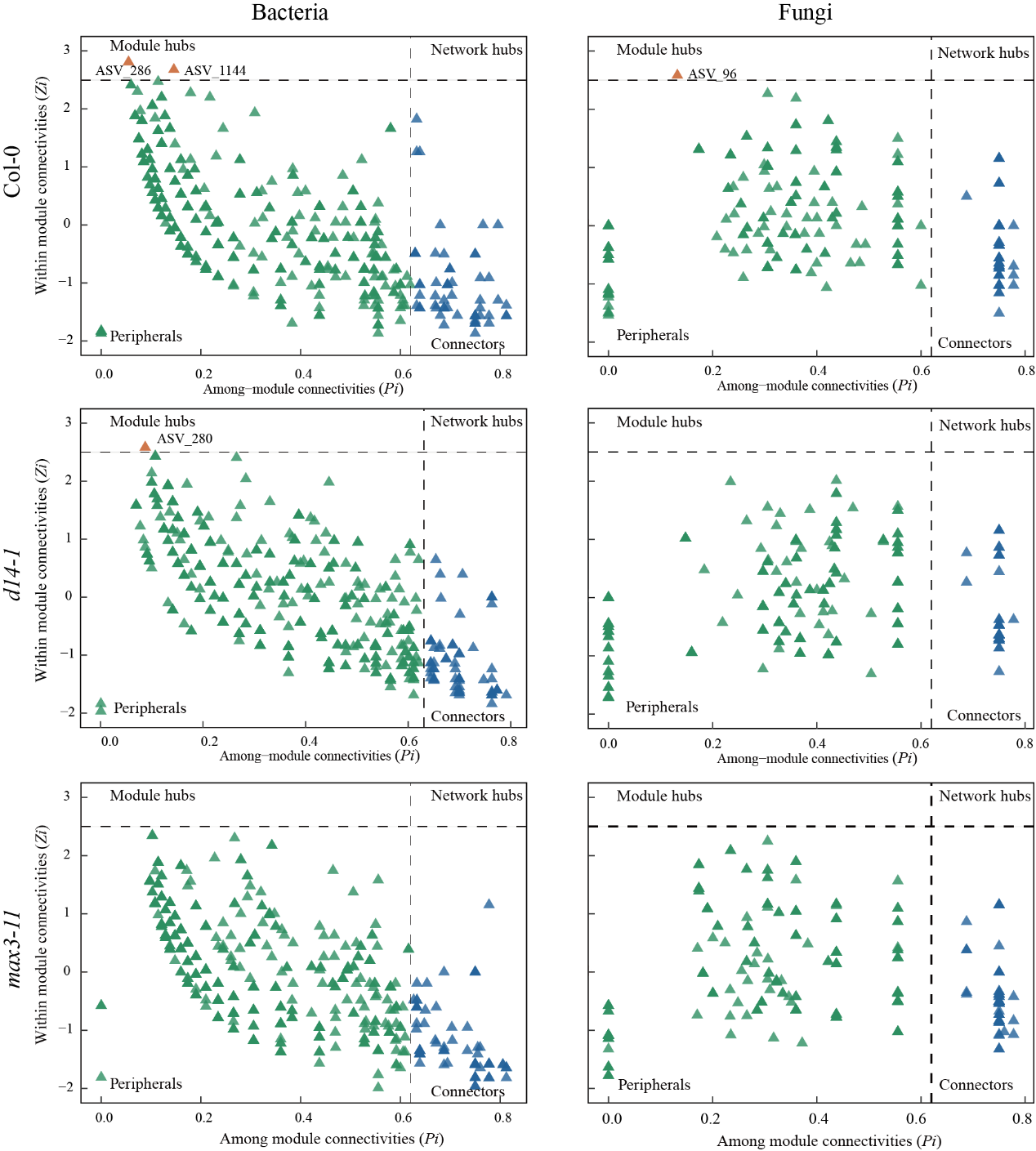

Supplement: Fig. S5 — Identification of keystone taxa. [file msystems.01124-23-s0005.tif]

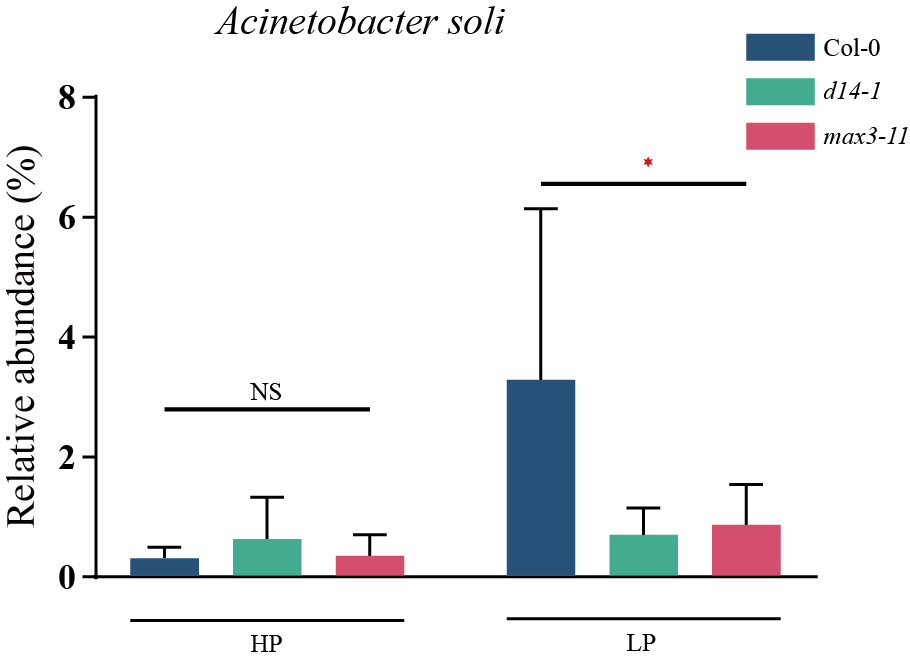

Supplement: Fig. S6 — Robustness. [file msystems.01124-23-s0006.tif]

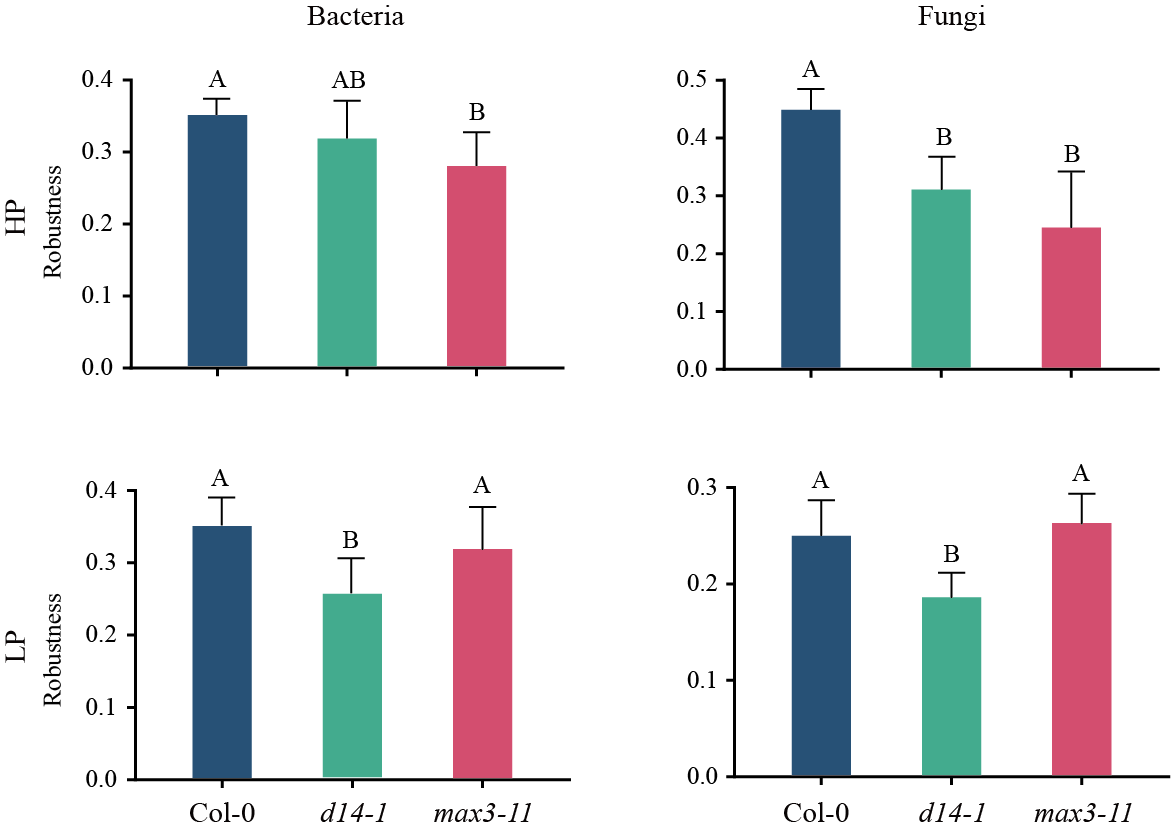

Supplement: Fig. S7 — Vulnerability. [file msystems.01124-23-s0007.tif]

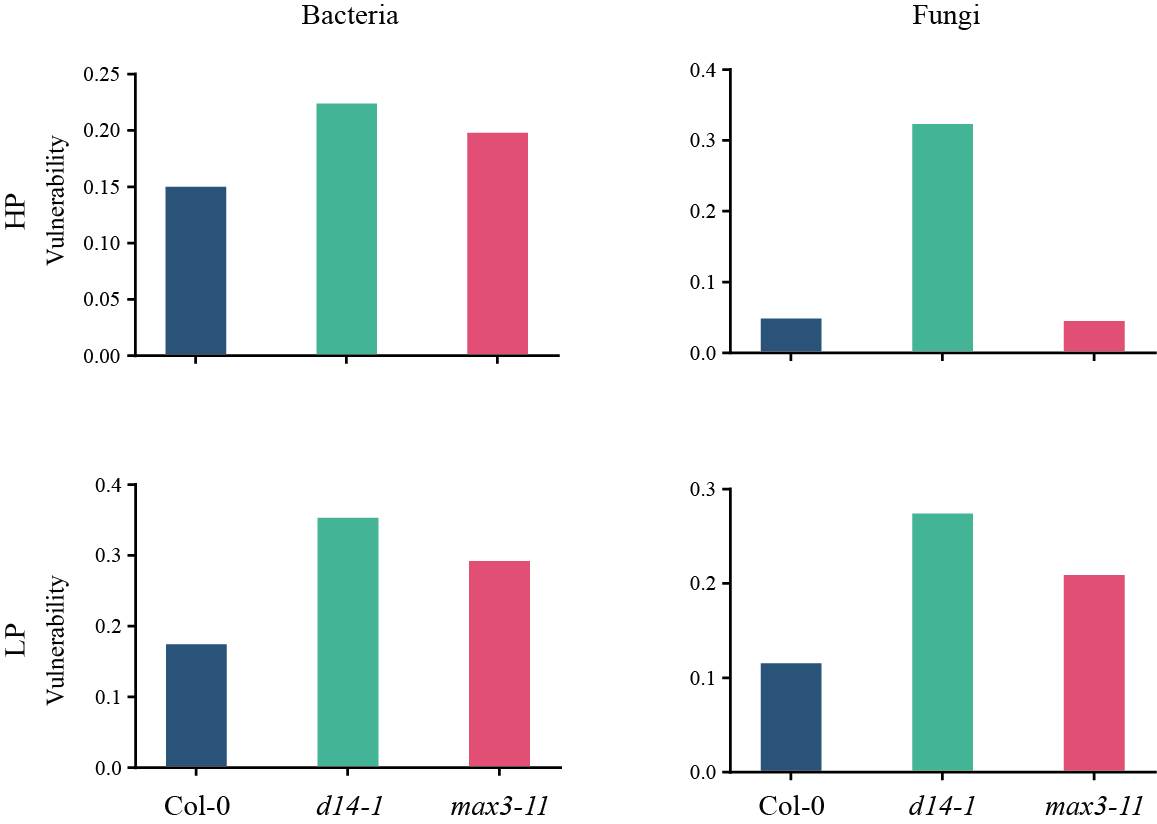

Supplement: Fig. S8 — Negative:positive cohesion. [file msystems.01124-23-s0008.tif]

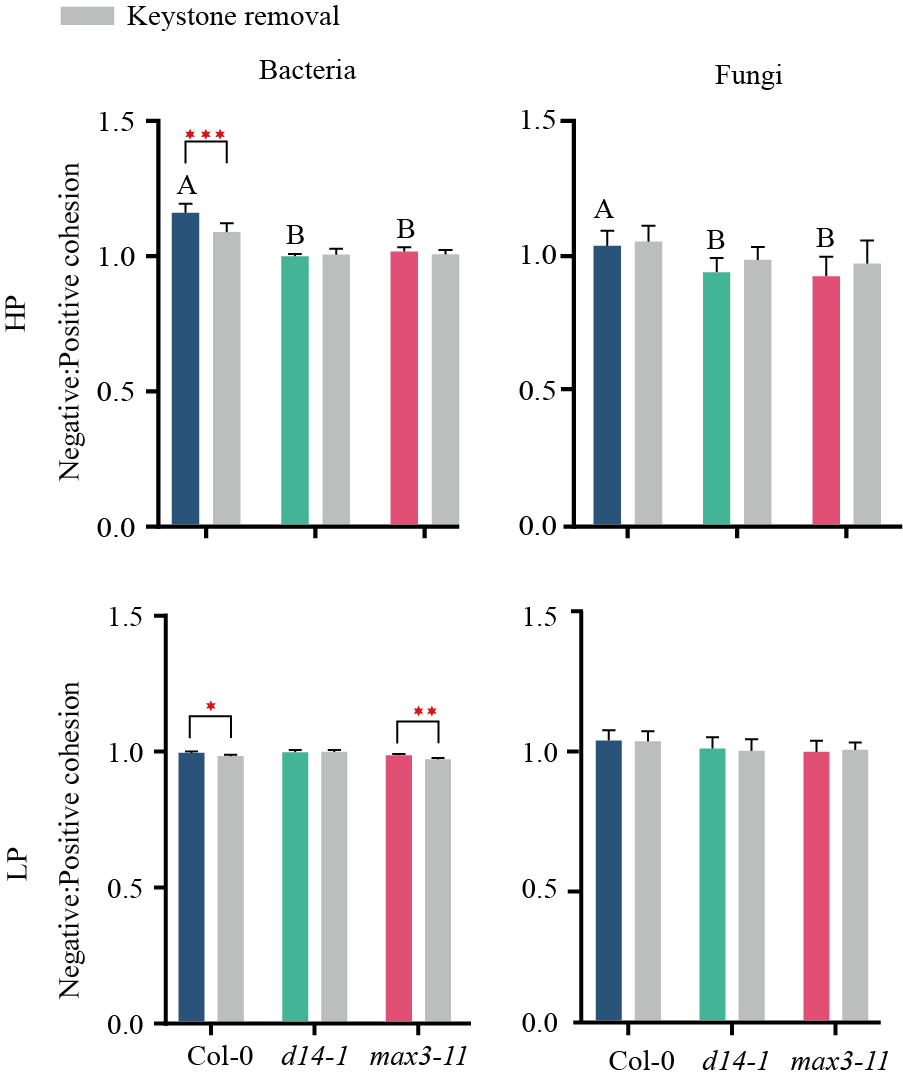

Supplement: Fig. S9 — Relative abundance of A. soli in rhizosphere microbial communities of A. thaliana. [file msystems.01124-23-s0009.tif]
