# Supplementary material for: Strigolactones shape the assembly of root-associated microbiota in response to phosphorus availability
Source: mSystems. 2024 May 23;9(6):e01124-23. doi: 10.1128/msystems.01124-23 (PMC11237589; doi:10.1128/msystems.01124-23)
Supplement: Supplemental material — Supplemental figure legends and tables. [file msystems.01124-23-s0010.docx]

**Strigolactones shape the assembly of root-associated microbiota in response to phosphorus availability**

Pubo Chen^1,2^, Pingliang Huang^1,2^, Haiyang Yu^1,2^, Huang Yu^3^, Weicheng Xie^1,2^, Yuehua Wang^1,2^, Yu Zhou^4^, Li Chen^1, 2, 3^, Meng Zhang^1, 2, 3^ ^*^, Ruifeng Yao^1, 2, 3^ ^*^

^1^State Key Laboratory of Chemo/Biosensing and Chemometrics, Hunan Provincial Key Laboratory of Plant Functional Genomics and Developmental Regulation, College of Biology, Hunan University, Changsha 410082, China

^2^Yuelushan Lab, Changsha, 410128, China

^3^Greater Bay Area Institute for Innovation, Hunan University, Guangzhou, 511300, China

^4^School of Resource & Environment and Safety Engineering, University of South China, Hengyang 421001, China

^5^Hunan Institute of Microbiology, Changsha 410007, China

^*^ Author for correspondence (e-mail zhangmeng2019@hnu.edu.cn, ryao@hnu.edu.cn)

**Figure legend**

**FIG S1** Response of the *Arabidopsis* plants under Pi starvation. (a) Arabidopsis seeds were germinated on 1/2-strength MS media for 7 days and then transplanted to either HP or LP media for another 21 days. (b) Data show mean shoot fresh weight ± SE (n = 6, n refers to the number of flowerpots (4 seedlings per flowerpot) per group). (c) Anthocyanins accumulation in shoots. (*P < 0.05; **P < 0.01, *** P < 0.001, **** P < 0.0001 one-way ANOVA).

**FIG S2** Activity of total acid phosphatases (a, b) and H_2_O_2_ concentrations (c, d) of whole plants in wild-type and *d14-1*, *max3-11* both under Pi-sufficient (HP) and Pi-deficient (LP) conditions for 12 days. (n=3, Different letters represent P < 0.05, one-way ANOVA)

**FIG S3** Relative abundances of the dominant phyla of rhizosphere and bulk soil microbial communities. Ternary plots of all OTUs detected in the rhizosphere of SL signaling plants.

**FIG S4** LDA scores of the abundant taxa with significant differences of bacteria, and fungi communities under HP (a,b) and LP (c,d) conditions, as identified using linear discriminant analysis (LDA) effect size (LEfSe).

**FIG S5** Identification of keystone taxa in wild type and SL mutant *d14-1*, *max3-11* under HP conditions based on their topological roles in networks. The within- (Zi) and among- (Pi) module connectivity plot was used to identify module (Pi < 0.62, Zi > 0.2.5) or network hub taxa (Pi > 0.62, Zi > 2.5), as well as connector (Pi > 0.62, Zi < 2.5) and peripheral taxa.

**FIG S6** Robustness is measured as the proportion of the remaining species in the community after random node 50% removal (Different letters represent P < 0.05, one-way ANOVA).

**Fig. S7** Vulnerability is measured as the maximum node vulnerability in each community.

**FIG S8** Negative: Positive cohesion is calculated from species abundances, columns with gray are calculated without keystone taxa. Different lowercase letters above the bars indicate significant differences. *P < 0.05; **P < 0.01, *** P < 0.001 (Student’s t-test)

**FIG S9** Relative abundance of *Acinetobacter soli* in rhizosphere microbial communities of *A. thaliana* under HP and LP conditions. (n=6, Student's t-test, *P < 0.05)

**TABLE S1** Physicochemical properties of local forest soil

| index | Minimum | Maximum | Mean value |
| --- | --- | --- | --- |
| pH | 4.84 | 5.27 | 4.99 |
| TC (g.kg^-1^) | 26.67 | 36.9 | 33.73 |
| TN (g.kg^-1^) | 3.22 | 3.81 | 3.49 |
| AP (mg.kg^-1^) | 6.82 | 8.42 | 7.64 |
| AK (mg.kg^-1^) | 59.75 | 70.9 | 65.46 |

**TABLE S2** Mean and standard deviation of alpha diversity measurement

| **HP (Mean** ± **Standard deviation, n=6)** | | | | | |
| --- | --- | --- | --- | --- | --- |
|  | Col-0 | *d14-1* | *max3-11* | soil |  |
| **16s** | | | | | |
| Shannon | 3.94 ± 0.14, ab | 3.85 ± 0.59, b | 3.84 ± 0.31, b | 4.40 ± 0.061, a |  |
| Chao1 | 1134.34 ± 95.55, ab | 982.04 ± 110.92, c | 1040.83 ± 102.50, bc | 1194.29 ± 36.69, a |  |
| Simpson | 0.079 ± 0.012, a | 0.109 ± 0.11, a | 0.082 ± 0.028, a | 0.046 ± 0.080, a |  |
| **ITS** |  |  |  |  |  |
| Shannon | 3.82 ± 0.18, a | 2.31 ± 0.43, b | 3.03 ± 0.60, a | 2.60 ± 0.38, ab |  |
| Chao1 | 237.78 ± 74.27, a | 196.82 ± 41.48, a | 228.46 ± 67.82, a | 186.58 ± 43.67, a |  |
| Simpson | 0.093 ± 0.026, bc | 0.28 ± 0.15, a | 0.12 ± 0.063, c | 0.15 ± 0.069, abc |  |
| **LP (Mean** ± **Standard deviation, n=6)** | | | | | |
|  | Col-0 | *d14-1* | *max3-11* | soil |  |
| **16s** | | | | | |
| Shannon | 5.07 ± 0.084, a | 4.69 ± 0.52, a | 4.83 ± 0.26, a | 5.14 ±0.24, a |  |
| Chao1 | 955.83 ± 35.24, ab | 796.32 ± 72.86, c | 898.25 ± 105.66, abc | 979.40 ± 97.14, a |  |
| Simpson | 0.020 ± 0.0030, a | 0.040 ± 0.039, a | 0.025 ± 0.0081, a | 0.019 ± 0.0084, a |  |
| **ITS** | | | | | |
| Shannon | 2.91 ± 0.25, a | 3.00 ± 0.37, a | 2.95 ± 0.50, a | 3.32 ± 0.40, a |  |
| Chao1 | 167.75 ± 33.35, a | 188.07 ± 24.16, a | 155.63 ± 22.08, a | 175.25 ± 39.08, a |  |
| Simpson | 0.13 ± 0.034, a | 0.12 ± 0.048, a | 0.15 ± 0.12, a | 0.09 ± 0.055, a |  |

**Table. S3** Topological properties of bacterial fungal community networks in HP and LP conditions

|  | 16s-HP | | | 16s-LP | | |
| --- | --- | --- | --- | --- | --- | --- |
| Network Indexes | Col-0 | *d14-1* | *max3-11* | Col-0 | *d14-1* | *max3-11* |
| nodes | 534 | 556 | 524 | 693 | 581 | 614 |
| edges | 1477 | 252 | 263 | 247 | 196 | 195 |
| Average degree | 5.53 | 0.91 | 0.98 | 0.72 | 0.66 | 0.63 |
| Average path length | 3.39 | 5.61 | 3.80 | 4.56 | 5.43 | 5.62 |
| Graph diameter | 10 | 14 | 11 | 12 | 13 | 15 |
| Graph density | 0.010 | 0.0016 | 0.0018 | 0.00060 | 0.0011 | 0.0010 |
| Clustering coefficient | 0.45 | 0.29 | 0.25 | 0.29 | 0.25 | 0.24 |
| Betweenness centralization | 0.011 | 0.017 | 0.016 | 0.0020 | 0.0077 | 0.0074 |
| Degree centralization | 0.12 | 0.020 | 0.060 | 0.0080 | 0.022 | 0.015 |
| modularity | 0.83 | 0.76 | 0.62 | 0.91 | 0.78 | 0.87 |
|  | ITS-HP | | | ITS-LP | | |
| nodes | 794 | 665 | 753 | 542 | 552 | 444 |
| edges | 576 | 234 | 357 | 279 | 246 | 296 |
| Average degree | 1.45 | 0.70 | 0.94 | 1.12 | 0.89 | 1.03 |
| Average path length | 7.44 | 2.35 | 3.21 | 2.27 | 2.94 | 2.12 |
| Graph diameter | 16 | 6 | 12 | 9 | 9 | 6 |
| Graph density | 0.0018 | 0.001 | 0.0012 | 0.0019 | 0.0016 | 0.003 |
| Clustering coefficient | 0.67 | 0.40 | 0.45 | 0.50 | 0.32 | 0.44 |
| Betweenness centralization | 0.018 | 0.0006 | 0.0016 | 0.0013 | 0.001 | 0.002 |
| Degree centralization | 0.027 | 0.024 | 0.026 | 0.037 | 0.029 | 0.046 |
| modularity | 0.86 | 0.81 | 0.77 | 0.84 | 0.84 | 0.81 |
